# Clinician’s opinion about critical risk results proposed by the Croatian Chamber of Medical Biochemists: a survey in one Croatian tertiary hospital

**DOI:** 10.11613/BM.2019.030711

**Published:** 2019-10-15

**Authors:** Pavica Šonjić, Ana Nikler, Dora Vuljanić, Lora Dukić, Ana-Maria Šimundić

**Affiliations:** 1 Department of medical biochemistry in oncology, University Hospital for Tumors, “Sestre milosrdnice” University Hospital Center, Zagreb, Croatia; 2 Department of Medical Laboratory Diagnostics, University Hospital “Sveti Duh”, Zagreb, Croatia

**Keywords:** critical results, survey, post-analytical phase, laboratory management

## Abstract

**Introduction:**

It has been recommended that each laboratory modify their critical result reporting practices to reflect the clinical needs of their patient populations. The aim of this survey was to assess how well critical laboratory values defined by the Croatian Chamber of Medical Biochemists (CCMB) correspond to the needs of the physicians at University hospital “Sveti Duh” (Zagreb, Croatia).

**Materials and methods:**

We conducted a survey among physicians from five departments in our hospital. Physicians were asked general questions about critical risk results (if and how they want to be informed). A list of critical risk results defined by the CCMB was offered and physicians were asked to revise the existing critical risk results and suggest adding new parameters. Obtained data were presented as numbers. Where the number of observations was low, ratios were used.

**Results:**

Survey response rate was 43% (52/121). Majority (48/52) wants to be informed of critical risk results, either personally (31/48) or through a colleague (32/48). They prefer to be informed about critical risk results of prothrombin time, platelet count, haemoglobin, glucose, creatinine, sodium and potassium. Revisions in the CCMB critical risk result list are proposed by 13 out of 48 physicians. Neonatologists approved the CCMB’s list.

**Conclusions:**

Although most critical risk results defined by the CCMB correspond well to the needs of the physicians in our hospital, some revisions are necessary to meet the particular needs of individual departments. Communication of critical risk results to those who have requested laboratory testing is highly appreciated practice.

## Introduction

The critical risk results of laboratory tests require urgent notification of the physician or other health-care workers responsible for the patient. These results indicate a life-threatening or major patient harm and require immediate medical action (*1*). Not all laboratory parameters are clinically equally important. Which laboratory tests and critical thresholds should be considered as important and necessary for urgent patient treatment is still the main debate topic of various studies (*2*-*4*). In a study conducted in 2014, harmonized terminology, policies and procedures for management of the critical risk results reporting were suggested (*4*). Critical risk results should be defined to ensure patient safety. In spite of initiatives for harmonization of critical risk results reporting, the lack of standardization is present among different laboratories worldwide (*5*, *6*).

The Croatian Chamber of Medical Biochemists (CCMB) established a document that encompasses the suggested parameters and their values of critical risk results (*7*). In 2015, the Croatian Society of Medical Biochemistry and Laboratory Medicine (CSMBLM) conducted the survey on critical results reporting in Croatian laboratories (*8*). They have shown that the majority of Croatian laboratories apply the CCMB’s recommendations. There are differences in reporting practices between hospital and general practice laboratories leading to the lack of standardization.

According to the Clinical and Laboratory Standards Institute (CLSI) GP47 guideline for Management of critical- and significant-risk results, each laboratory should modify their policy to reflect the clinical needs of their patient populations because no single approach applies to every health care environment (*9*). In line with this recommendation, we have undertaken this study to understand how well current critical laboratory values defined by the CCMB correspond to the needs of the physicians in University hospital “Sveti Duh” (Zagreb, Croatia).

## Materials and methods

### Study design

This survey was conducted among physicians at University hospital „Sveti Duh“ (Zagreb, Croatia), from May to November 2018. Included physicians were from Department of Internal medicine (included departments: Department of Gastroenterology and Hepatology, Department of Clinical Pharmacology and Toxicology, Department of Clinical Immunology, Rheumatology and Pulmonology, Department of Nephrology and Dialysis, Department of Emergency and Intensive Care), Department of Anaesthesiology, Reanimation and Intensive Care, Department of Neurology, Department of Surgery and Department of Neonatology.

Survey was formed as anonymous questionnaire consisting of 5 questions (Table 1). Given the differences in critical risk results for new-borns and adults, two forms of questionnaire were designed. One form was for the Department of Neonatology and another for other departments. Questionnaires in the form of leaflets were distributed to physicians.

**Table 1:** The questionnaire form

**Questions**				
1. Specify the clinic or department where you work:				
2. Do you want to be informed of critical risk results:				
a) YES b) NO				
3. Who can be informed of critical risk results?				
a) I want to be informed personally b) another physician from the department/clinic can be informed c) nurse from the department/clinic can be informed d) others				
4. A table with the list of critical risk results defined by the Croatian Chamber of Medical Biochemists (CCMB) is shown. In left column mark parameters for which critical risk results you want to be informed. Where you think it’s needed, in right column write down revision of cut-off for specific critical risk result.				
If you want to be informed of critical risk result, mark the parameter	Parameter	Critical risk results (defined by the CCMB)	Suggestion of different cut-off for critical risk result	What was your revision based on? (scientific literature, national guidelines, own experience, …)
5. Do you suggest any other parameter not listed in the previous table whose critical risk result is important?				
a) YES b) NO If your answer is affirmative, please write down what parameters do you suggest and its critical risk result.				

The first section of the questionnaire included three questions, with the third one offering multiple choice statements. Physicians were asked to specify their department. They had to define if they want to be informed of critical risk results and whom they want to be reported about critical risk results. Second section was designed as a table with a list of critical risk results defined by the CCMB. Participants were asked to select parameters for which they want to be notified about critical risk results. They were also asked to write down suggestion for revision of cut-off for specific critical risk result. If a suggestion for revision was offered, they were asked to give a rationale. In the last section physicians were offered to suggest parameter that wasn’t presented in the table, together with appropriate critical risk result.

### Statistical analysis

Data were presented as numbers. Where number of observations was low, ratios were used. Statistical analysis was performed using Microsoft Office Excel 2007 (Microsoft Corporation, Redmond, Washington, USA).

## Results

The overall response rate in this study was 43% (52 out of total number of physicians from all included departments, N = 121). Response rates for each included department are presented in Table 2. Out of total number of responded surveys (N = 52), 48 physicians stated that they want to be notified about critical risk results for certain parameters. Out of these 48 physicians, 4 were from the Department of Neonatology.

**Table 2:** Response rates for each department included in the survey

**Department**	**Response rate**
Department of Internal medicine	11/28
Department of Anaesthesiology, Reanimation and Intensive Care	22/41
Department of Neurology	9/12
Department of Surgery	6/34
Department of Neonatology	4/6
All departments	52/121
The response rate is presented as number of physicians participating in the survey/total number of physicians in the department.	

In the first section of the survey, 31/48 participants reported that they wish to be personally notified about critical risk results, 32/48 stated that the information can be transmitted to another physician in the department and 22/48 stated that critical risk results can be communicated to the nurses. Only 6 out of 48 physicians suggested that information about critical risk results could be reported in other ways - depending on the situation or through Laboratory Information System (LIS).

The proportion of physicians who want to be reported about critical risk results of certain parameters are presented in Table 3. Prothrombin time (PT), haemoglobin and platelets were selected for notification by most of the physicians (31, 28 and 29 respectively out of 44). Regarding biochemistry parameters, physicians found most important critical risk results of glucose (29/44 for low and 27/44 for high result), creatinine (25/44), sodium (27/44 for low result) and potassium (32/44 for both critical risk results). On the other hand, only few physicians want to be notified on phosphorus (8/44 for low and 6/44 for high result), magnesium (9/44 for high result) and antithrombin (9/44) critical risk results. All 4 paediatricians from the Department of Neonatology indicated critical risk results of C-reactive protein, haemoglobin, haematocrit, potassium and leukocytes, approving values defined by the CCMB. When asked to suggest critical risk result which they thought to be more suitable than those defined by the CCMB, only 13 out of 48 physicians gave their proposals. Revised and suggested critical risk results, along with medical specialty and physicians’ rationales are shown in Table 4. Paediatricians from the Department of Neonatology have not revised any critical risk results for new-born infants.

**Table 3:** The number of physicians who want to be informed about critical risk results

**Parameter**	**Critical risk results (defined by the CCMB)**	**Frequency of answers,** **N (proportion)**
Activated partial thromboplastin time (APTT)	75 s	20 (0.45)
Prothrombin time (PT)	< 0,15; INR ≥ 5	31 (0.70)
Antithrombin (AT3)	< 50%	9 (0.20)
Fibrinogen	< 0.8 g/L	18 (0.41)
Leukocytes	< 2 x 10^9^/L	25 (0.57)
	> 38 x 10^9^/L	20 (0.45)
Haemoglobin	< 66 g/L	28 (0.64)
	> 199 g/L	19 (0.43)
Haematocrit	< 0.180 (L/L)	22 (0.50)
	> 0.610 (L/L)	15 (0.34)
Platelets	< 20 x 10^9^/L	29 (0.66)
Amylase (serum)	>350 U/L	16 (0.36)
Lipase	> 700 U/L	14 (0.32)
Aminotransferases (AST, ALT)	> 1000 U/L	22 (0.50)
Creatine kinase (CK)	> 1000 U/L	17 (0.39)
Lactate dehydrogenase (LD)	> 500 U/L	14 (0.32)
Glucose	< 2.5 mmol/L	29 (0.66)
	> 27.8 mmol/L	27 (0.61)
Urea	> 35.6 mmol/L	22 (0.50)
Creatinine	> 654 μmol/L	25 (0.57)
Uric acid	> 773 μmol/L	12 (0.27)
Bilirubin	> 257 μmol/L	16 (0.36)
Sodium	< 120 mmol/L	27 (0.61)
	>160 mmol/L	24 (0.55)
Potassium	< 2.8 mmol/L	32 (0.73)
	> 6.0 mmol/L	32 (0.73)
Chloride	< 75 mmol/L	11 (0.25)
	> 125 mmol/L	10 (0.23)
Calcium (total)	< 1.65 mmol/L	15 (0.34)
	> 3.50 mmol/L	15 (0.34)
Calcium (ionized)	< 0.78 mmol/L	16 (0.36)
	> 1.60 mmol/L	15 (0.34)
Inorganic phosphorus	< 0.32 mmol/L	8 (0.18)
	>2.9 mmol/L	6 (0.14)
Magnesium	< 0.41 mmol/L	11 (0.25)
	> 2.00 mmol/L	9 (0.20)
Ammonia	> 59 μmol/L	20 (0.45)
Lactate	> 5.0 mmol/L	15 (0.34)
Digoxin	> 2.0 μg/L	20 (0.45)
Ethanol	> 3.5 g/L	16 (0.36)
**Parameter**	**Critical risk results (defined by the CCMB)**	**Frequency of answers,** **N (proportion)**
pO2	< 5.7 kPa	22 (0.50)
pCO2	< 2.5 kPa	19 (0.43)
	> 6.7 kPa	19 (0.43)
pH	< 7.200	22 (0.50)
Free T4	> 45 pmol/L	12 (0.27)
C-reactive protein (CRP)	> 5.0 mg/L	4 (1.00)*
Glucose	< 1.8 mmol/L	0*
	> 18.2 mmol/L	0*
Haematocrit	< 0.330 (L/L)	4 (1.00)*
	> 0.710 (L/L)	4 (1.00)*
Haemoglobin	< 85 g/L	4 (1.00)*
	> 230 g/L	4 (1.00)*
Potassium	< 2.6 mmol/L	4 (1.00)*
	>7.7 mmol/L	4 (1.00)*
Leukocytes	< 5.0 x 10^9^/L	4 (1.00)*
	> 25.0 x 10^9^/L	4 (1.00)*
pO2	< 4.9 kPa	0*
Platelets	< 100 x10^9^/L	0*
CCMB – Croatian Chamber of Medical Biochemists. Total number of physicians from other departments N = 44. Total number of physicians from the Department of Neonatology N = 4. *frequency of answers of physicians from the Department of Neonatology.		

**Table 4:** Critical risk results proposals and rationales provided by physicians of different specialties

**Parameter and critical risk result defined by the CCMB**	**Critical risk results suggested by physicians**	**Specialty** **(N = number of physicians)**	**Rationale**
Platelets (20 x10^9^/L)	< 30 x10^9^/L	gastroenterology (N = 1)	/
	< 50 x10^9^/L	anaesthesiology (N = 2)	/
	< 60 x10^9^/L	anaesthesiology (N = 1)	for delivery room
	< 80 x10^9^/L	anaesthesiology (N = 3)	guidelines for invasive procedures
	100 x10^9^/L	anaesthesiology (N = 1)	/
Haemoglobin (< 66 g/L)	< 70 g/L	gastroenterology (N = 1)	/
		anaesthesiology (N = 1)	according to literature
	80 g/L	anaesthesiology (N = 1)	/
	< 90 g/L	anaesthesiology (N = 1)	guidelines for patients with comorbidity
Haemoglobin (> 199 g/L)	>170 g/L	anaesthesiology (N = 1)	/
Fibrinogen (< 0.8 g/L)	< 1 g/L	gastroenterology (N = 1)	/
	< 1.5 g/L	anaesthesiology (N = 3)	for delivery room
	2.0 g/L	anaesthesiology (N = 1)	/
PT (< 0.15)	0.2	gastroenterology (N = 1)	/
Leukocytes (> 38 x10^9^/L)	> 20 x10^9^/L	gastroenterology (N = 1)	/
Glucose (< 2.5 mmol/L)	< 3.0 mmol/L	anaesthesiology (N = 2)	/
	< 3.0 mmol/L	pharmacology (N = 1)	based on experience
Glucose (> 27.8 mmol/L)	> 20 mmol/L	pharmacology (N = 1)	based on experience
	> 25 mmol/L	gastroenterology (N = 1)	/
		anaesthesiology (N = 1)	/
Creatinine (> 654 μmol/L)	> 500 μmol/L	pharmacology (N = 1)	based on experience
		gastroenterology (N = 1)	/
	> 300 μmol/L	anaesthesiology (N = 1)	/
Urea (> 35.6 mmol/L)	> 30 mmol/L	pharmacology (N = 1)	based on experience
		gastroenterology (N = 1)	/
Amylase (> 350 U/L)	> 500 U/L	gastroenterology (N = 1)	/
AST, ALT (> 1000 U/L)	> 500 U/L	gastroenterology (N = 1)	/
CK (> 1000 U/L)	> 500 U/L	gastroenterology (N = 1)	/
Bilirubin (> 257 μmol/L)	> 200 μmol/L	gastroenterology (N = 1)	/
Potassium (< 2.8 mmol/L)	< 3.0 mmol/L	anaesthesiology (N = 1)	/
Potassium (> 6.0 mmol/L)	6.2 mmol/L	anaesthesiology (N = 1)	/
	6.5 mmol/L	anaesthesiology (N = 1)	indication for renal replacement therapy
Ammonia (> 59 μmol/L)	> 80 μmol/L	gastroenterology (N = 1)	/
pCO_2_ (> 6.7 kPa)	> 7.0 kPa	anaesthesiology (N = 1)	/
CCMB – Croatian Chamber of Medical Biochemists. PT - prothrombin time. AST - aspartate aminotransferase. ALT - alanine aminotransferase. CK - creatine kinase. / - no rationale was provided for critical risk result proposal.			

Majority of physicians, who want to be reported about critical risk results, had no suggestions of other parameters that weren’t included in the attached table. Only two physicians indicated D-dimer (proposed critical risk result > 1500 µg/L) as a parameter they would like to be reported about. Moreover, there were 6 physicians who stated that they want to be informed about critical risk results, but they have not answered any other question nor marked parameters in following table.

## Discussion

This survey reveals the attitude and needs of physicians for critical risk results in our institution. Results showed that most of physicians want to be informed of critical risk results defined by the CCMB, specifically of those referring to PT, platelet count, haemoglobin, glucose, creatinine, sodium and potassium. These parameters are also found in critical risk result lists of laboratories participating in survey conducted in the United States by Howanitz *et al.* However, they report that parameters and results included in the lists vary widely among participants (*5*). Lippi *et al.* also reported variable list of critical risk results among surveyed Italian laboratories (*10*). The most common reported parameters on their lists include electrolytes, glucose, haemoglobin, platelet count, arterial blood gases, PT and activated partial thromboplastin time. Glucose, creatinine, sodium and potassium are also found in reported critical risk results list for biochemical parameters in the UK study by Tillman *et al.* (*11*). Interestingly, UK laboratories presented differences in reported results depending on the person that is being informed (physician or nurse).

In our survey, proposed critical risk results for the most revised haematological parameters (*e.g.* platelets, haemoglobin) were less extreme than the ones in use. It suggests that physicians want stricter limits, so they could intervene more promptly. We suppose these revisions are based on their long-time experience in differences in patient’s treatments and professional guidelines. Contrary to our results, Salinas *et al.* reported that critical risk results used in their routine laboratory are: 10 x10^9^/L for platelet count and < 50 g/L for haemoglobin, which is substantially lower than what was suggested by our participants, and even lower than critical risk result from the CCMB’s list (*12*, *13*). Their critical risk result is based on agreement with hospital cardiologists, rheumatologists, endocrinologists and haematologists. On the other hand, in a Brazilian survey by Torres *et al.* lower critical risk result for platelets was set at < 20 x10^9^/L through a consensus of the cardiac emergency department and cardiac intensive care unit physicians (*14*). This value corresponds to the one on the CCMB’s list.

Proposed critical risk results for upper limit of potassium are higher than the one on the CCMB’s list (> 6.0 mmol/L), which is similar to the results reported in the United States by Howanitz *et al.* and in Spain by Salinas *et al.* (*5*, *13*). Since proposed limits are less strict and appear on other critical risk result lists, we can assume that the surveyed physicians base their proposal on professional experience.

Although D-dimer is not included in the CCMB’s critical risk results list, few physicians from our survey want to be reported on that parameter. Proposed critical risk result represents an increased concentration of D-dimer that can occur in wide range of clinical conditions (*15*). Negative result for the group of patients with a low pre-test probability can be used to rule out deep vein thrombosis (DVT) or pulmonary embolism (PE), while positive result does not confirm DVT and PE and thus requires additional testing to confirm diagnosis (*16*). It would be interesting to understand rationale behind this request. Nevertheless, this was beyond the scope of this survey.

Revision of paediatric critical risk result list confirmed that some critical risk results have more importance than others. Similar survey was conducted in Canada to review already tailored paediatric list of 26 critical risk results of 12 parameters (*17*). Parameters included in both lists are potassium, bilirubin, glucose and pO_2_. They all differ in critical risk results, while also potassium, bilirubin and glucose on Canadian list are presented with more critical risk results depending on child age. Canadian list was revised and updated in agreement with paediatricians from their institution and national guidelines. For instance, bilirubin has four critical risk results for neonates during their first four days. All results are higher than the one presented in CCMB’s list (> 239 μmol/L), but they are changed in accordance with guidelines on neonatal hyperbilirubinemia published by their national paediatric society. Critical risk result for low arterial pO_2_ on their list is higher than ours (> 4.9 kPa). Interestingly, they reported different responses for revision of this parameter between intensivists and general paediatricians, with intensivists proposing more extreme critical risk results. Paediatric population is also included in a Brazilian survey of Torres *et al.* (*14*). In comparison with ours, their list includes more parameters. It is a common list for adults and paediatrics, with some of the critical risk results modified for the paediatric population. Their list includes all parameters from ours, except for arterial pO_2_. They highlight that pO_2_ is a part of laboratory’s priority flow, whose result is reported before other analyses are done. Since their list is based on the IFCC document as is the CCMB’s list, common parameters have the same values (*3*). Exceptions are bilirubin and potassium. Critical risk result for bilirubin is kept the same as the one for adult population on the CCMB’s list. On the other hand, critical risk results for potassium are different than ours and adjusted to relevant literature from the field by Goyal *et al.* (*18*). They set potassium critical risk results to < 3.5 mmol/L and > 5.5 mmol/L.

Consistent with our results, a survey conducted in Canada revealed similar attitudes regarding organization of critical risk results reporting (*19*). Most of their physicians also prefer to be notified personally about critical risk results. The same situation is reported in Italy in survey by Lippi *et al.*, showing that critical risk results are mainly notified to physicians (*10*). There are surveys that confirmed positive effects of prompt critical risk result notification which led to change in patient’s treatment (*5*, *14*, *20*). By tailoring list of critical risk results in agreement with physicians, better patient care is provided, less unnecessary alerts are made, and laboratory has better post-analytical management.

There are some limitations of this survey. Firstly, moderate overall response rate and unsatisfactory low response rate for particular departments (*e.g.* Department of Surgery). Also, data collected in this survey are not transferable to other laboratories, because it shows the needs of our physicians and particular patient population in our hospital. Therefore, further surveys including more participating physicians, higher response rates and more departments are still needed. Nevertheless, we still believe that our results provide a valuable insight into heterogeneity of physicians’ attitudes about critical risk results and the limitations of the ones currently used in accordance with the CCMB’s list.

In conclusion, physicians in our hospital reported that they want to be informed of critical risk results. Although they mostly accept critical risk results proposed by the CCMB, some of them have suggested new cut-offs for current critical risk results, as well as adding new parameter. This confirms the need of defining our own list in accordance with the needs of individual departments for prompt and adequate clinical response. This survey sets basis for organizing critical risk reporting procedure.
